# A practical guide to the diagnosis and management of suspected Non-tuberculous Mycobacterial Pulmonary Disease (NTM-PD) in the United Kingdom

**DOI:** 10.1038/s41533-024-00403-9

**Published:** 2024-12-21

**Authors:** D. J. Dhasmana, P. Whitaker, R. van der Laan, F. Frost

**Affiliations:** 1 Victoria Hospital, Kirkcaldy, NHS Fife, Kirkcaldy, UK; 2 Infection and Global Health, School of Medicine, University of St Andrews, St Andrews, UK; 3 Bradford Teaching Hospitals, Bradford, UK; 4 Insmed Incorporated, Bridgewater, NJ USA; 5 Liverpool Heart & Chest Hospital NHS Foundation Trust, Liverpool, UK; 6 Institute of Infection, Veterinary and Ecology Sciences, University of Liverpool, Liverpool, UK

**Keywords:** Respiratory tract diseases, Diagnosis, Therapeutics

## Abstract

Non-tuberculous Mycobacterial Pulmonary Disease (NTM-PD) is a chronic disease characterised by progressive inflammatory lung damage due to infection by non-tuberculous mycobacteria (NTM). Global prevalence of NTM-PD is generally low but is rising, likely due to a combination of increased surveillance, increasing multimorbidity and improved diagnostic techniques. Most disease is caused by *Mycobacterium avium* complex species. NTM-PD can be challenging to both diagnose and manage but given the risk of untreated disease and the challenges around drug treatments, it is vital that all healthcare professionals involved in primary care consider NTM-PD at the earliest opportunity. In particular, NTM-PD should be considered where there are respiratory symptoms in the setting of pre-existing chronic lung disease such as chronic obstructive pulmonary disease (COPD) and bronchiectasis. Early suspicion should lead to appropriate primary screening measures. This article discusses the relevance of NTM-PD today, risk factors for developing disease, pathways from clinical presentation to referral to specialist care, and discusses management and drug treatments. A flow diagram of a screening process is presented as a guideline for best practice from a United Kingdom perspective.

## Introduction

Non-tuberculous mycobacteria (NTM) represent a large group of Mycobacteria that are separate to *Mycobacterium tuberculosis* complex and *Mycobacterium leprae*. They are ubiquitous in natural and artificial environments but can manifest as opportunistic lung pathogens when inhaled in NTM-loaded aerosols or dust particles^1–3^. Environmental sources include domestic plumbing such as shower aerosols, drinking water distribution systems (through biofilm formation), hot tubs and spas; and natural sources such as soil, peat, brown-water swamps, and boreal forestry. Exposure can also occur in clinical settings. For example, *M. chimaera* infection has been observed following open cardiac surgery with cardiopulmonary bypass machines, in which contaminated heater-cooler units were implicated^4^.

**Fig. 1:**
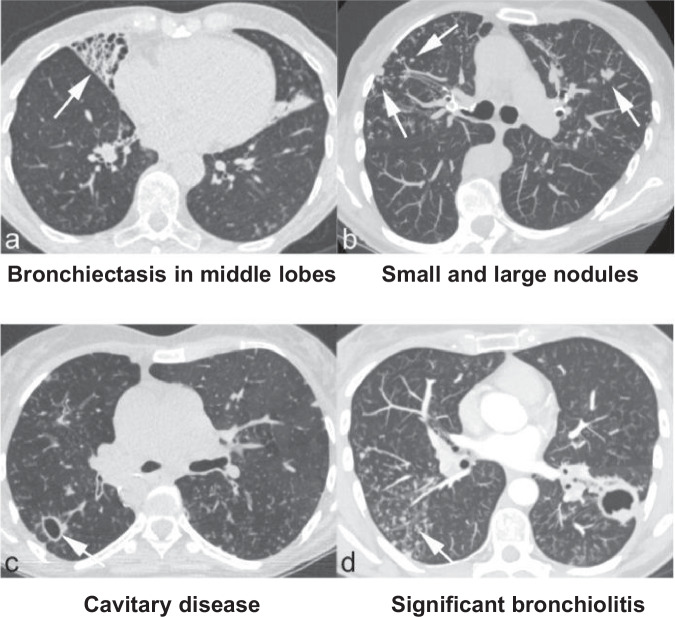
Typical computed tomography scans in patients with non-tuberculous mycobacterial pulmonary disease. **a** Bronchiectasis in middle lobes; **b** small and large nodules; **c** cavitary disease; **d** significant bronchiolitis. Reproduced under Creative Commons BY license from Dettmer S, et al. (2021) J Clin Med. 10(12).

Although NTM can cause disease in other organs, pulmonary disease is the most common setting for serious illness^5^. NTM may be cultured from the lung incidentally without any pathological or symptomatic sequalae. However, when this is associated with symptoms and characteristic imaging, NTM pulmonary disease (NTM-PD) should be considered. NTM-PD has two major subtypes^3^. The first is nodular bronchiectatic disease, previously known as ‘Lady Windermere syndrome’ (present in both men and women), which manifests as bronchiectasis with small nodules and a 'tree-in-bud' appearance on high-resolution computed tomography (HRCT). The second is fibrocavitary disease, which may appear similar to tuberculosis with large fibrotic cavities, typically forming in the upper lobes of the lungs. In some cases, localised disease can mimic lung cancer on CT and positron emission tomography-CT imaging, and a tissue biopsy may be the first time the diagnosis is considered^6^. Fig. 1 shows some typical CT findings in patients with NTM-PD^7^.

Nearly 200 species of NTM have been identified and are generally classified as either slow-growing or rapid-growing^3,8^. *Mycobacterium avium* complex (MAC) represents a group of slow-growing species including its most prominent members, *M. avium*, *M. intracellulare* and *M. chimaera*^5,9^. Infection with these species is the most common cause of NTM-PD. *Mycobacterium abscessus* is an example of a ‘rapid-grower’ usually associated with a more aggressive disease course, especially in cystic fibrosis (CF) or in severely immunocompromised patients^10^.

NTM-PD is a growing global concern, with increasing prevalence and significant challenges associated with both its diagnosis and management. In this article, we discuss the growing relevance of NTM-PD, consider those patients most at risk of NTM infection, describe the most useful initial diagnostic tests, and discuss reasons to refer to specialist care, including key issues around management and drug treatments.

### Burden of NTM-PD

NTM-PD prevalence is ~6.5 per 100,000 in European countries but appears to be rising in many geographical regions globally^11^, at least in part due to increased surveillance, awareness, and better access to diagnostic techniques. It is predicted to rise further with advancing medical treatments, increased immune suppression, and rising multimorbidity^1,12,13^. A study in the United Kingdom (UK) reported increased prevalence in recent years^14^ with MAC responsible for most of this increase^15,16^. In a 2013 analysis of NTM from European pulmonary samples in 2008, the UK had the second-highest percentage of MAC isolates at 44% of all NTM species^16^.

Co-existing lung diseases including bronchiectasis, chronic obstructive pulmonary disease (COPD) and CF are common amongst patients with NTM-PD^1,15^. Chronic respiratory disease was associated with a 16.5-fold increase in risk of NTM-PD, which was even higher in those receiving high-dose inhaled steroid. In a smaller number of cases, NTM-PD is the primary insult and causes significant lung damage, typically localised bronchiectasis. Immune suppressive states including cancer, primary and secondary immunodeficiencies, the latter commonly including Acquired Immunodeficiency Syndrome (AIDS), and profound immune suppressive treatments such as cancer chemotherapy, anti-tumour necrosis factor (TNF) therapy, and immunosuppressive treatment post solid organ transplant all lead to a significantly increased risk of NTM infection and therefore disease^17^. NTM-PD is usually seen in those with chronic respiratory disease, whilst extrapulmonary NTM is observed almost exclusively with immune suppression or traumatic inoculation (e.g. cosmetic surgery with inadequate sterilization/wound management)^18^.

NTM-PD is associated with significantly reduced health-related quality of life^19^. Patient-reported outcomes (PROs) may also predict worse outcomes^20^. NTM-PD can be progressive and sometimes contribute to mortality, when untreated MAC lung disease results in extensive cavitary lung destruction and chronic respiratory failure^5^. A recent meta-analysis of mortality in 17 studies of high-risk patients with MAC-PD found that five-year overall all-cause mortality was 27% (95% confidence interval 21.3–33.0%), with a range of 5–42%^21^. Patients with fibrocavitary disease are at higher risk of mortality than those with nodular bronchiectatic disease^22,23^. Survival time may also differ according to the NTM species^23,24^.

Alongside the morbidity and mortality associated with untreated NTM-PD sit considerable unmet needs and potentially serious consequences. These include the delayed initial diagnosis of NTM-PD, treatment failure^25^, antibiotic resistance^26^, refractory disease^27^, and high rates of relapse (same species and strain) or re-infection (different species or strain)^28^. Delayed diagnosis often results from uncertainty regarding significance of a first positive NTM culture. A key early issue, therefore, is the healthcare professional’s appreciation that a patient’s symptoms may represent NTM-PD in the first place. Certain risk factors may lower the threshold for further assessment and the appropriate diagnostic tests.

### Risk factors for NTM-PD

Several established genetic risks are associated with susceptibility to NTM-PD. Patients with mutations in immune-related genes can be more susceptible^1,29,30^. Mutations of genes involved in mucociliary clearance can prevent normal cilia development or result in thickening of mucus through dysfunctional or absent ion channels, as is apparent with the CF transmembrane conductance regulator (CFTR) in CF. Indeed, CFTR dysfunction may drive significant disease in those without a formal diagnosis of CF. Further studies on association, cause and even treatments are in discussion^31,32^. Variations in connective tissue genes may also influence susceptibility^29^.

There is a specific body type associated with nodular bronchiectatic disease in post-menopausal women, including a lean, tall stature, skeletal abnormalities such as scoliosis and low body mass index (BMI)^33^. The well-documented description of ‘Lady Windermere syndrome' referring to nodular bronchiectasis in this context is challenged in an article that considers confounding underlying abnormalities, including CFTR dysfunction, connective tissue disease and dural ectasia behind the body phenotype^34^. In contrast, risk factors for fibrocavitary disease include male sex (particularly late-middle aged men), cigarette smoking, excessive alcohol use and structural lung disease (e.g. COPD)^5,35^. In recent years there appears to be a shift from fibrocavitary disease in middle-aged men who smoke to the nodular bronchiectatic form of disease in older women^12^. In a study focused on NTM-PD related hospital admissions in Germany, traditional risk factors associated with more severe disease were confirmed, including COPD, emphysema, smoking, malnutrition, and bronchiectasis^36^. Other studies across the world reviewing national datasets generally observe the same risk factors described above, also noting use of steroids (either inhaled or systemic), previous tuberculosis, age, and interstitial lung disease^37–39^.

A study from the USA highlighted that socioeconomic and environmental factors also play a part, including high population density, higher education and income, and high surface water levels^40^. Other research has reported associations between the risk of developing NTM-PD and employment in heavy industry, lower socioeconomic status, and air pollution^41^, which may partially explain the global species diversity^16^. Some of these risks and the mechanisms behind their influence on NTM infection remain unclear.

### Clinical presentation

In addition to the risk factors and patient characteristics outlined above, those active in primary and secondary care settings should be aware of the symptoms associated with NTM infection. Symptoms of NTM-PD are frequently respiratory but usually non-specific and shared by patients with co-morbid conditions such as COPD and bronchiectasis, especially with increasing age and advanced disease. Chronic cough, usually productive of sputum (occurring almost every day in 25% and 44% of patients respectively in one study^42^), and shortness of breath are the commonest symptoms, with non-respiratory symptoms including lethargy, fatigue, anorexia, and malaise^43^. Additional symptoms may include weight loss, haemoptysis, chest pains, sweats, and fever^44^.

It is particularly important to consider NTM in patients with systemic symptoms and pre-existing lung disease. Radiological features in CT scans may suggest NTM infection and typically include cavities, nodules, and ‘tree-in-bud’ appearances reflecting small airway involvement through ‘exudative bronchiolitis’. However, these changes are not diagnostic or specific, and NTM-related imaging is a complex area with significant variation in interpretation^7,45,46^. In a cohort of patients with bronchiectasis and NTM-PD, there was a higher prevalence of bronchiectasis in the middle lobes, and CT patterns differed from those patients with only bronchiectasis^7^. Older female patients with higher Medical Research Council (MRC) dyspnoea scores and bilateral lingula and middle lobe involvement are more likely to have bronchiectasis and NTM-PD^47^. Plain chest x-rays are unlikely to be diagnostic in themselves with only non-specific features visible, if at all. However, they may detect cavities which should raise suspicion of NTM and prompt urgent onward referral. Given their value, chest x-rays would always be recommended in patients with suspected NTM infection.

### Referral and diagnosis

Once an initial positive NTM culture has been identified, patients should be discussed with secondary care for further assessment. If significant symptoms and imaging features are present, then the typical next step would be a formal review in the specialty clinic. If the culture finding was incidental or unexpected, repeat sampling and a discussion may be more appropriate in the first instance.

The key clinical question at this point is whether the first positive culture is coincidental to the patient’s health or whether it represents NTM-PD and a reason for the observed respiratory symptoms. The secondary care assessment should take into account the clinical, microbiological (number and source of positive cultures) and radiological features of the patient’s condition in deciding if this may represent NTM-PD and therefore whether treatment should be offered^48^.

In the UK, patients with NTM-PD are identified through various settings, including respiratory clinics, emergency hospital admissions, and lung cancer multidisciplinary teams/fast-track referrals. Fewer are identified from screening in primary care. Most patients with a new positive culture are referred to the local respiratory service for further assessment unless they have known complex disease such as CF or bronchiectasis and are already receiving specialist respiratory care.

Guidelines from the UK British Thoracic Society (BTS)^48^ agree on the need for the three major components of clinical features to be present, composed of symptoms, microbiology (positive cultures), and radiology (consistent imaging features on CT) to diagnose NTM-PD. Microbiological criteria include ≥2 sputum samples, or a single positive culture from biopsy or a bronchoalveolar lavage. Therefore, referral to secondary care should be simultaneously supported with ≥2 further requests for sputum mycobacteria culture. Radiological criteria are more difficult to define with certainty; consistent imaging features include nodular or cavitary structures on chest radiograph, nodular bronchiectasis or fibrocavitary appearances against a background of bronchiectasis on CT. During these investigations, it is important to assess for alternative or contributing causes of the presenting symptoms, including *Pseudomonas aeruginosa, Aspergillus spp*., tuberculosis (TB) and lung cancer^3,48^. The NTM UK network is currently exploring a structured radiology reporting process which could improve recognition of new disease and management of known NTM-PD on treatment^49^.

**Fig. 2:**
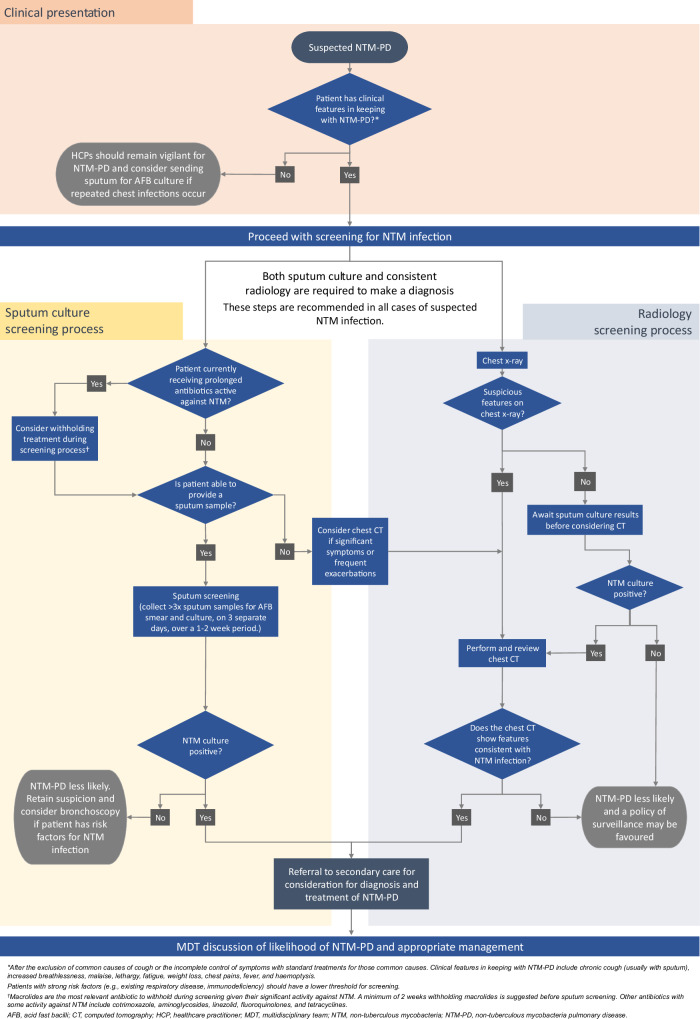
Flowchart of recommended screening process for patients with suspected non-tuberculous mycobacterial pulmonary disease.

Fig. 2 illustrates potential routes from suspicion of NTM-PD at clinical presentation to diagnosis, as outlined by the guidelines described above.

European Respiratory Society (ERS) and BTS guidelines both recommend mycobacterial culture in patients with bronchiectasis where NTM are suspected as an aetiological cause of the disease, or where patients have radiological or clinical features of NTM infection. The guidelines also recommend consideration of routine annual surveillance for NTM in patients with bronchiectasis^48,50^.

Guidelines on diagnosis and management of other respiratory conditions also exist that consider screening for NTM infection. Cystic fibrosis guidelines developed by NICE (National Institute of Clinical Excellence) recommend formal annual reviews that include sputum cultures for NTM^49^. NICE guidelines on the management of COPD are less specific, only advising sputum culture prior to initiation of antibiotics, without additional recommendations on screening for NTM, specifically^51^.

In the UK, initial laboratory sputum reports usually return ‘Mycobacterium culture positive’ results, pending differentiation of TB and NTM by the reference laboratory. Local tests are often available in-house and primary care physicians should consider the relative suspicion of TB versus NTM. Rapid polymerase chain reaction (PCR) tests for TB can be requested if a sputum sample has a high bacterial load and is positive by acid-fast bacillus (AFB) smear microscopy, (i.e. ‘smear-positive’), or if clinical suspicion of TB is high. Some services routinely test all smear-positive samples for TB. TB PCR tests offer a high positive predictive value, thus positive PCR makes TB very likely and NTM-PD very unlikely. A negative TB PCR is not strongly predictive of a lack of TB infection, especially if the initial sputum smear is negative. Sputum samples with lower mycobacterial loads will be smear-negative but remain under culture in the local microbiology labs for up to 8 weeks before a final report is issued. Where the culture becomes positive, the lab report will issue a ‘positive culture’ interim report, pending final species identification results.

Currently, no commercially available rapid PCR assays for NTM species exist and advanced molecular methods are often required to accurately identify NTM species, although there is now a UK-wide intention to move to whole genome sequencing (WGS) to rapidly and definitively speciate^52–54^. WGS is rapid, comprehensive and may provide information on drug sensitivity, and is therefore ultimately likely to be cost-effective in service delivery. Matrix-assisted laser desorption ionisation-time of flight mass spectrometry (MALDI-TOF MS) is an alternative diagnostic platform more economical than sequencing and may have a place in NTM diagnostics^55^.

It is important to identify the NTM species to stratify risk in terms of NTM-PD, and to guide initial treatment strategy. Some species are highly likely to be pathogenic and clinically relevant, for example, those in the *Mycobacterium avium* complex. Others, e.g. *Mycobacterium gordonae*, are much less likely to be relevant for pulmonary disease. Different species may also require different treatment regimens. In the case of recurrent infection, it may be important to recognise whether this represents relapse or reinfection. WGS would address this question, further advocating for its UK-wide roll-out.

Diagnosing NTM-PD can be challenging and patients with NTM-positive cultures may be incorrectly diagnosed^56^. Primary care professionals should be supported in having a better understanding of NTM-PD to enable more rapid diagnosis. This is particularly relevant amongst those professionals managing high-risk patients as described above. A multidisciplinary approach is encouraged to confidently diagnose NTM-PD^57^ and access to expertise in specialist centres may be required to accurately interpret scans or laboratory results. A consensus diagnosis should be reached where uncertainty exists. The patient must remain aware of any discrepancies or uncertainties around their diagnosis.

### Treatment of NTM-PD

NTM-PD treatment is highly challenging and involves multi-drug regimens for extended periods of time. The most commonly used antimycobacterial drugs include macrolides, ethambutol, rifamycins, and aminoglycosides (Table 1). Amikacin Liposome Inhalation Suspension (ALIS, or Arikayce liposomal) is approved for treatment of MAC-PD in patients with refractory disease, whilst new trials are ongoing on its potential use in earlier settings (NCT04677569)^58^. Given that the duration of antibiotic therapy is the time to culture conversion plus a further 12 months, regimens typically last 18 months or more. Alongside the risks of side effects^3,59,60^, treatment regimen duration and complexity impose a significant treatment burden on already comorbid patients.

**Table 1:** Antibiotic regimen summaries for the treatment of the most common species^48^.

MAC	
	R, E and C/Az (systemic Am should be considered in cases of severe cavitary disease) Addition of amikacin liposome inhalation suspension is recommended in cases of refractory MAC (patients who have failed therapy after ≥6 months of guideline-based treatment)^3^.
*M. abscessus*	
	Initial phase: Minimum 4-week course of Am and tigecycline (both IV), and (where tolerated) intravenous imipenem. Oral C/Az should be used (where tolerated), except in cases of constitutive macrolide resistance. In addition, 1–3 more oral agents should be considered, selected from clofazimine, linezolid, and possibly others^2,3^. Continuation phase: Nebulised Am and a macrolide (oral Az or C), with 1–3 of the following: clofazimine, linezolid, minocycline or doxycycline, moxifloxacin, or ciprofloxacin, and co-trimoxazole (guided by drug susceptibility and patient tolerance). In cases of inducible macrolide resistance, nebulised Am should be used with 2–4 of the following: clofazimine, linezolid, minocycline or doxycycline, moxifloxacin, or ciprofloxacin, and co-trimoxazole.
*M. kansasii*	
	In cases of Rifampicin sensitivity, R, E and I or a macrolide (C/Az). In cases of Rifampicin resistance, a three-drug regimen guided by drug susceptibility test results.
*M. malmoense*	
	RE and I or a macrolide (C/Az). Injectable aminoglycoside (Am or S) should be considered in cases of severe disease. Nebulised Am could be considered as an alternative to intra-venous/muscular administration.
*M. xenopi*	
	Four-drug regimen (where tolerated) comprising R, E, and a macrolide (C/Az), with either a quinolone (ciprofloxacin or moxifloxacin) or isoniazid. Injectable aminoglycoside (Am or S) should be considered in cases of severe disease.

*Am* amikacin, *Az* azithromycin, *C* clarithromycin, *E* ethambutol, *I* isoniazid, *IV* intravenous, *R* rifampicin, *S* streptomycin.

Guidelines for treatment based on the best available evidence have been updated by the BTS in 2017, the American Thoracic Society/Infectious Diseases Society of America/ ERS/European Society of Clinical Microbiology and Infectious Diseases in 2020, and by the United States Cystic Fibrosis Foundation/European Cystic Fibrosis Society (2016)^3,5,48,61^. Poor adherence to guideline-based treatment (GBT) can result in worse outcomes for patients and may promote antibiotic resistance^52,62^. GBT remains strongly advocated across international societies in this context, until ongoing trials can provide a greater evidence base for change and improved benefits over harms^63^.

Treatment decisions are influenced by many factors (Table 2) and best managed by a multidisciplinary team^57^, including a respiratory physician, respiratory nurse, physiotherapist, infectious disease physician, microbiologist, radiologist and pharmacist. Pharmacists form a vital component of treatment and help to manage and advise regarding drug-drug interactions and toxicity inherent to multidrug regimens. Untreated NTM-PD has been associated with disease progression and poorer prognosis^64^, particularly in high-risk patients^65^. Radiological progression has been demonstrated in over half of untreated patients^52^. The risk-benefit ratio may change over time, as the danger of disease progression overcomes the burden and risks of treatment^66^.

**Table 2:** Key factors to consider before initiating treatment for NTM-PD.

Disease type and severity	
	How pathogenic is the isolated NTM species?
	Is the sputum sample smear-positive (representing a higher load of bacteria)?
	Is the disease fibrocavitary or nodular bronchiectatic?
	What is the risk of disease progression?
	Have all pre-existing conditions including treatment of any other infections been optimised?
Treatment burden issues	
	Does the treatment burden (in particular, the drug tolerance and toxicity) outweigh the disease burden?
	Is the patient willing and able to commit to multidrug treatment for an extended period?
	Is the patient willing and able to commit to the requirements for monitoring of drug treatments e.g., ECG, audiometry, regular bloods, sputum culture?
	Are there predictable and serious drug-drug interactions? Can they be managed safely with monitoring or drug changes?
Additional potential therapies	
	Is there a potential role for surgical resection of the worst affected areas, or adjunctive therapies? Rarely, those with specific immune deficiencies may be suitable for specific immune support or immunomodulatory therapy^70^.

*ECG* electrocardiogram, *NTM* non-tuberculous mycobacteria.

For patients established on treatment, regular multidisciplinary team review is required, sputum cultures should be obtained every 1–2 months to assess treatment response in the early period, and advice given on good practice around airway clearance and nutrition. If a patient remains culture-positive 6 months after treatment, an important marker of refractory disease, then a management strategy that includes alternative drug treatment regimens should be carefully considered. In these patients, there is evidence of greater lung function decline and radiological progression^67,68^. In refractory but localised disease, there may be a role for surgical resection in selected cases.

Even after successful treatment, NTM-PD recurrence is high, in some studies almost 50% of patients^28,69^. Recurrence should be considered in all patients who become symptomatic with a history of prior NTM-PD. Most recurrences, particularly in patients with nodular-bronchiectatic disease, relate to reinfection with underlying environmental and immunological factors likely to blame, whilst increased relapse of disease in cavitary disease is likely related to drug penetration and immune function^69^.

## Conclusion

NTM-PD is associated with significant disease and treatment burdens, the latter in terms of both treatment side effects and duration. With NTM infection rates increasing, awareness and suspicion of possible NTM-PD should be commonplace among healthcare professionals, particularly those managing patients with pre-existing lung disease and high-risk factors including malnutrition and immune suppression. Healthcare professionals in primary and secondary care settings managing patients with risk factors for NTM-PD should be aware of relevant screening procedures and referral pathways to enable timely assessment and diagnosis. Treatment should be guideline-based, involve multidisciplinary teams with expert consultation, and with a patient fully informed of both the goals and the risks of treatment to achieve the best outcomes overall.

### Reporting summary

Further information on research design is available in the Nature Research Reporting Summary linked to this article.

## Supplementary information

Reporting Summary

## Data Availability

Data sharing is not applicable to this article as no datasets were generated or analysed during the current study.
