# Publisher Correction: A practical guide to the diagnosis and management of suspected Non-tuberculous Mycobacterial Pulmonary Disease (NTM-PD) in the United Kingdom

**DOI:** 10.1038/s41533-025-00439-5

**Published:** 2025-07-05

**Authors:** D. J. Dhasmana, P. Whitaker, R. van der Laan, F. Frost

**Affiliations:** 1 Victoria Hospital, Kirkcaldy, NHS Fife, Kirkcaldy, UK; 2 Infection and Global Health, School of Medicine, University of St Andrews, St Andrews, UK; 3 Bradford Teaching Hospitals, Bradford, UK; 4 Insmed Incorporated, Bridgewater, NJ USA; 5 Liverpool Heart & Chest Hospital NHS Foundation Trust, Liverpool, UK; 6 Institute of Infection, Veterinary and Ecology Sciences, University of Liverpool, Liverpool, UK

**Keywords:** Respiratory tract diseases, Diagnosis, Therapeutics

Correction to: *npj Primary Care Respiratory Medicine* 10.1038/s41533-024-00403-9, published online 21 December 2024

“In the original version of the article, the legend to Figure 1 should have read ‘Typical computed tomography scans in patients with non-tuberculous mycobacterial pulmonary disease’. This has now been corrected in the PDF and HTML versions of the article".

